# Symptom Profiles of Children and Young People 12 Months after SARS-CoV-2 Testing: A National Matched Cohort Study (The CLoCk Study)

**DOI:** 10.3390/children10071227

**Published:** 2023-07-14

**Authors:** Snehal M. Pinto Pereira, Manjula D. Nugawela, Kelsey McOwat, Emma Dalrymple, Laila Xu, Shamez N. Ladhani, Ruth Simmons, Trudie Chalder, Olivia Swann, Tamsin Ford, Isobel Heyman, Terry Segal, Malcolm G. Semple, Natalia K. Rojas, CLoCk Consortium, Roz Shafran, Terence Stephenson

**Affiliations:** 1 Division of Surgery & Interventional Science, Faculty of Medical Sciences, University College London, London WC1E 6BT, UK; 2 UCL Great Ormond Street Institute of Child Health, University College London, London WC1N 1EH, UK; 3 Immunisations and Vaccine Preventable Diseases, UK Health Security Agency, 61 Colindale Avenue, London NW9 5EQ, UK; 4 Paediatric Infectious Diseases Research Group, St. George’s University of London, Cranmer Terrace, London SW17 0RE, UK; 5 Department of Psychological Medicine, Institute of Psychiatry, Psychology and Neuroscience, King’s College London, De Crespigny Park, London SE5 8AF, UK; 6 Centre for Medical Informatics, Usher Institute, University of Edinburgh, Edinburgh EH16 4TL, UK; 7 Department of Psychiatry, Hershel Smith Building Cambridge Biomedical Campus, University of Cambridge, Cambridge CB2 0SZ, UK; 8 University College London Hospitals NHS Foundation Trust, London NW1 2PG, UK; 9 NIHR Health Protection Research Unit in Emerging and Zoonotic Infections, Department of Clinical Infection, Microbiology and Immunology, Institute of Infection, Veterinary and Ecological Sciences, University of Liverpool, Liverpool L69 3BX, UK; 10 Respiratory Medicine, Alder Hey Children’s Hospital, Liverpool L12 2AP, UK

**Keywords:** post-COVID-19 condition, long COVID, children and young people, non-hospitalised, matched cohort study

## Abstract

Background: Although 99% of children and young people have been exposed to SARS-CoV-2, the long-term prevalence of post-COVID-19 symptoms in young people is unclear. The aim of this study is to describe symptom profiles 12 months after SARS-CoV-2 testing. Method: A matched cohort study of a national sample of 20,202 children and young people who took a SARS-CoV-2 PCR test between September 2020 and March 2021. Results: 12 months post-index-test, there was a difference in the number of symptoms reported by initial negatives who never tested positive (NN) compared to the other three groups who had at least one positive test (*p* < 0.001). Similarly, 10.2% of the NN group described five-plus symptoms at 12 months compared to 15.9–24.0% in the other three groups who had at least one positive test. The most common symptoms were tiredness, sleeping difficulties, shortness of breath, and headaches for all four groups. For all these symptoms, the initial test positives with subsequent reports of re-infection had higher prevalences than other positive groups (*p* < 0.001). Symptom profiles, mental health, well-being, fatigue, and quality of life did not vary by vaccination status. Conclusions: Following the pandemic, many young people, particularly those that have had multiple SARS-CoV-2 positive tests, experience a range of symptoms that warrant consideration and potential investigation and intervention.

## 1. Introduction

In March 2020, patient advocacy groups reported that COVID-19 survivors were often left with persisting health problems rather than making a complete recovery. This phenomenon of COVID-19 “long-haulers” is also referred to as Long COVID-19 and post-acute sequelae of COVID-19 [1]. Here, we use World Health Organisation terminology: post-COVID-19 condition (PCC). By Autumn 2020, persisting health problems after SARS-CoV-2 infection were also being reported in children and young people [2] despite SARS-CoV-2 being a less severe infection [3,4] compared to adults [5,6,7].

Perhaps naively, in those early days of the pandemic, it seemed it might be reasonably straightforward to characterise this new condition: identify children and young people who had been infected, match them to an uninfected control group, follow both groups over time, describe the excess health problems in the infected group and analyse whether and how fast that excess declined over time. If only researching during a pandemic with a new virus was so simple. Imagine undertaking a comparable methodological approach with a hepatitis virus. Determining infection would be easy; symptoms such as jaundice or bleeding would be causally attributable to the virus; liver function tests would act as objective biomarkers; finding an uninfected matched, control group would be straightforward. In contrast, children and young people were being infected with SARS-CoV-2 before testing became widely available. Symptoms (other than loss of taste and smell) were not specific, and no consistent PCC biomarker has been identified, and, with successive pandemic waves and new viral strains, there is no longer any uninfected matched, control group. In addition, vaccination after infection and/or onset of PCC further confuses the picture, as does the phenomenon that after any catastrophe, survivors can experience the onset of new and chronic health problems. Hence, the debate of the ‘long pandemic’ versus PCC arises [8].

The children and young people in the Long COVID-19 (CLoCk) study is the largest national, matched longitudinal cohort study of children and young people in England [9]. In CLoCk, children and young people self-report their post-COVID-19 health after laboratory-confirmed SARS-CoV-2 infection. Bearing in mind the methodological challenges outlined above, this longitudinal study comprising SARS-CoV-2 PCR-positive and matched PCR-negative children and young people do allow us to describe the prevalence of health problems in children and young people aged 11–17 years old from across England who underwent PCR testing between September 2020–March 2021 (baseline) and, as a ‘snap-shot’, 12 months after testing. We have also published, using online Delphi consensus methodology [10], a research definition of PCC. This is defined as a condition occurring in children and young people with a history of confirmed SARS-CoV-2 infection, with at least one persisting symptom for a minimum duration of 12 weeks after initial testing that cannot be explained by an alternative diagnosis. The symptoms have an impact on everyday functioning, may continue or develop after COVID-19 infection, and may fluctuate or relapse over time. We have operationalised this definition using CloCk study data [9]. 

In previous CloCk study publications, the test-positive children and young people have been compared to laboratory-confirmed SARS-CoV-2 test-negative children and young people [9]. However, given that by June 2022, 82.0% of 5–11-year-olds and 99.3% of 12–18-year-olds had SARS-CoV-2 antibodies [11], in this publication, we no longer compare test-positive to test-negative children and young people. Instead, we report on follow-up at 12 months on four groups of children and young people: ‘initial test-negatives with no subsequent positive test’ (NN); ‘initial test-negatives with a subsequent positive test’ (NP); ‘initial test-positives with no report of subsequent re-infection’ (PN); and ‘initial test-positives with subsequent report of re-infection’ (PP).

**Hypothesis** **1** **(H1).** *All four infection status groups would have some symptoms 12 months after initial testing.*

**Hypothesis** **2** **(H2).** *The most common symptoms would be tiredness, sleeping difficulties, shortness of breath, and headaches [12]*.

**Hypothesis** **3** **(H3).** *The PP group would have more symptoms than the two other positive groups (i.e., NP and PN).*

**Hypothesis** **4** **(H4).** *Symptoms would not differ by vaccination status.*

## 2. Materials and Methods

The CLoCk study is described in detail elsewhere [9]. Briefly, CLoCk is a cohort study of SARS-CoV-2 PCR-positive and PCR-negative children and young people, PCR-tested between September 2020 and March 2021. The PCR-positive children and young people, aged 11–17 years, were matched by month of test, age at last birthday, sex, and geographical area (based on lower super output area) to SARS-CoV-2 test-negative children and young people using the SARS-CoV-2 testing dataset held by United Kingdom Health Security Agency (UKHSA, formally known as Public Health England). These were the only variables in the dataset available for matching. After obtaining informed consent, children and young people filled in an online questionnaire about their health at the time of their SARS-CoV-2 PCR test (“baseline”; retrospectively reported) and at approximately 3, 6 and 12 months after their index-PCR test (with different numbers of respondents at each time point depending on the time of recruitment into the study relative to their test date). 

**Figure 1:**
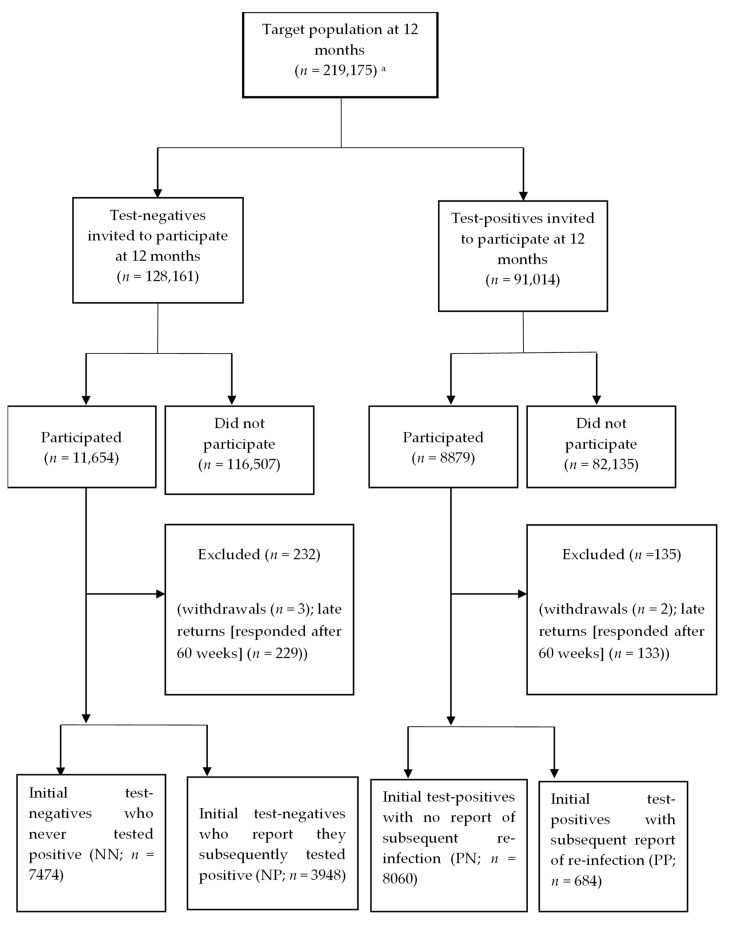
Participant Flow Diagram^;^ Footnote: classification into the 4 groups accounts for PCR test information held at UKHSA and also self-report. ^a^ target population includes those who were first contacted at 3, 6 and 12 months post-index PCR test, i.e., the only children and young people who could respond at 12 months were those who registered at 3 months (*n* = 7356) or 6 months (*n* = 10,530) or those approached for the first time at 12 months (*n* = 91,281).

The dataset held by UKHSA can track repeated testing longitudinally within individuals. In addition, we ask children and young people if they ever tested positive for SARS-CoV-2 (by either PCR or Lateral Flow Tests). Hence, the children and young people were divided into four categories: ‘initial test-negatives with no subsequent positive test’ (NN); ‘initial test-negatives with a subsequent positive test’ (NP); ‘initial test-positives with no report of subsequent re-infection’ (PN) and ‘initial test-positives with subsequent report of re-infection’ (PP), see Figure 1 for details.

### 2.1. Measures

The CLoCk questionnaire included demographics, elements of the International Severe Acute Respiratory and emerging Infection Consortium (ISARIC) Paediatric COVID-19 questionnaire [13], the recent Mental Health of Children and Young People in England surveys [14] and originally included 21 symptoms (mostly assessed as present/absent) [15]. The validated health scales: the Strengths and Difficulties Questionnaire (SDQ) [16], Short Warwick Edinburgh Mental Wellbeing Scale (SWEMWS) [17], Chalder Fatigue Scale [18], and the EQ-5D-Y as a measure of quality of life and function [19] were also included. The SDQ has good internal consistency (mean Cronbach α: 0.73), cross-informant correlation (mean: 0.34), and retest stability after 4-to-6 months (mean: 0.62) [16]. The SWEMWBS is a short version of the Warwick–Edinburgh Mental Wellbeing Scale (WEMWBS) with robust psychometric properties. It has been validated for use in young people [20,21], and while the test–retest reliability of SWEMWBS has not been reported for most populations [22], the WEMWBS test-retest reliability within 7–8 days after first completion was moderate in a UK population of 13-to-16-year-olds [23]. The Chalder Fatigue Scale has reliability coefficients that are high in studies of chronic fatigue syndrome patients [24] as well as in occupational and general population research, ranging from 0.90 to 0.83 [25]. Finally, the EQ-5D-Y, based on the EQ-5D-3L [26], consists of a descriptive system that comprises five dimensions: mobility, looking after self, doing usual activities, having pain/discomfort, and feeling worried, sad, or unhappy. Each dimension has 3 levels: no problems, some problems, and a lot of problems, and the instrument is feasible, reliable, and valid [27]. 

The CLoCk questionnaire was largely unchanged between initial and subsequent follow-ups: redundant questions (e.g., demographics, symptoms at time of testing etc.) were removed at follow-ups, and questions on additional symptoms (e.g., sleeping difficulties) were added [16]. Partway through data collection at 12 months (i.e., only for those with index-test January 2021-March 2021; *n* = 621), a symptom severity scale, a symptom impact scale, and questions on the type of COVID-19 vaccination received and date each dose was administered were added.

### 2.2. Statistical Methods

To assess representativeness of study participants, we compared their demographic characteristics (sex, age, region of residence, and Index of Multiple Deprivation) to the target population (i.e., all those targeted to take part in CLoCk by 12 months post-index-test). To determine the symptom profile of all four infection status groups 12 months after initial testing (objective 1), we describe the total number of symptoms (0,1, 2, 3, 4, and 5+) reported, prevalence of individual symptoms, and self-rated health 12 months post-index-test. We assessed symptom severity and impact 12 months post-index-test in the subsample of children and young people for whom we collected this data. Examining the prevalence of individual symptoms, we visually determine whether the most common symptoms were tiredness, sleeping difficulties, shortness of breath, and headaches (objective 2). We assessed prevalence of meeting the PCC research definition [10] 12 months post-index-test by infection status. In CLoCk, PCC was operationalized as having at least 1 symptom and experiencing some/a lot of problems with respect to mobility, self-care, doing usual activities or having pain/discomfort, or feeling very worried/sad. For the NN group, the requirement of a positive test was excluded so that comparisons could be made to the PN group. To meet the criteria for the research definition of PCC, 12 weeks need to have passed since confirmation of a positive test. We were unable to confirm with certainty the last date of a positive test in the NP, and PP groups, so we could not differentiate with confidence acute vs. long-term COVID-19 symptoms; hence, the prevalence of PCC in these groups was not determined. We examined study participant characteristics at 12 months post-index-test (including validated scales) stratified by infection status and PCC status at 12 months. We use chi-squared or Mann—Whitney tests to determine if symptom profiles at 12 months post-index-test differed between the PP group and the NP and PN groups (objective 3). We also determined whether hospital attendance and overnight stay during the 12-month period differed between groups. Finally, study participant characteristics on the validated scales, 12 months post-index-test were stratified by infection status groups and vaccination status at 12 months (objective 4). 

Ethical approval was provided by the Health Research Authority (REC reference: 21/YH/0060; IRAS project ID: 293495). 

## 3. Results

### 3.1. Sample Characteristics

We included 20,202 children and young people in our analytic sample out of 219,175 in the target population (response rate: 9.2%; Figure 1). These children and young people were largely similar to the target (Table 1), albeit with proportionately more responses from females, the least deprived, the East of England, and fewer from the West Midlands. Twelve-month follow-up questionnaires were returned at a median of 52.8 weeks (inter-quartile range: 51.4–55.1) after the index-PCR test. Prevalence of vaccination by 12 months was 76–79% for the NN and PN groups and 62–67% for the NP and PP groups (Supplementary Table S1), albeit we are unable to determine the chronology of (re)infection dates in relation to vaccination dates in these latter two groups. 

### 3.2. Symptoms 12 Months Post-Index-Test by Infection Status

The most striking finding was that 50.0% of the NN group had some symptoms compared to 61.3% (PN), 67.5% (NP), and 74.1% (PP) for the other three groups (pchi2-test < 0.001). Likewise, only 10.2% of the NN group described five-plus symptoms 12 months post-index-test compared to 16.0% (PN), 19.7% (NP), and 24.0% (PP) (Supplementary Table S2). Median self-rated overall health (scored from 0 [worst] to 100 [best]) was generally high (85–90) 12 months post-testing for all infection status groups (although there were statistical differences between the four groups: p-Kruskal–Wallis test = 0.001; Supplementary Table S2). Similarly, in the sub-sample of children and young people with information on symptom severity (*n* = 621), the median score in all four groups was 50; for symptom impact (*n* = 618), the median score ranged from 40 to 50 in all four groups (scale for both severity and impact from 0 (not severe/no impact) to 100 (extremely severe/impacting)).

### 3.3. Specific Symptoms, Quality of Life/Function, and PCC 12-Months Post-Index-Test 

**Figure 2:**
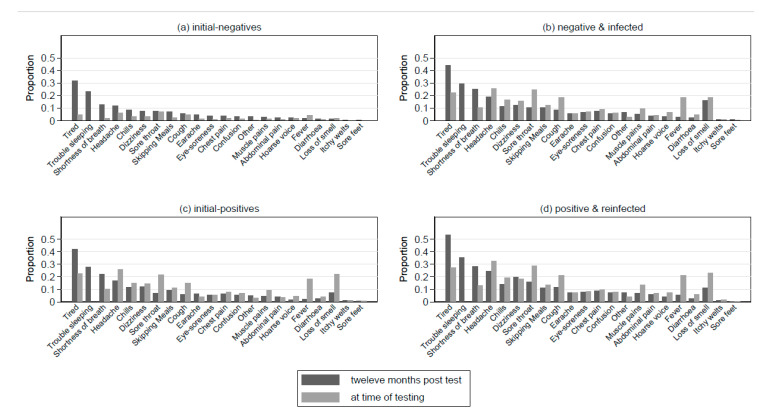
Proportion of participants experiencing symptoms at time of index test (reported retrospectively) and twelve months post-testing among (**a**) initial-negatives, (**b**) negatives and infected, (**c**) initial-positives, and (**d**) positives and reinfected. Note: sleeping difficulties included only at 12 months post-index-test, therefore data were not available at time of testing.

The specific symptoms experienced most commonly at the 12-month follow-up for all four groups were tiredness, sleeping difficulties, shortness of breath, and headaches (Figure 2a–d). Loss of smell was present 12 months post-testing in 7.2% (PN), 11% (PP), and 16.2% (NP) for those who had ever had a positive test but only in 1.4% for the NN group (pchi2-test < 0.001). 

**Figure 3:**
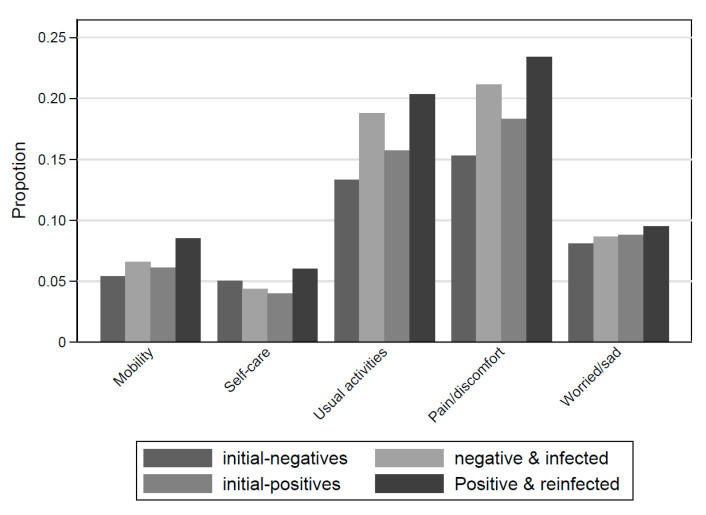
Proportion of participants reporting “some or a lot of problems” 12-month post-index-test by SARS-CoV-2 status. Note: Reporting some or a lot of problems with mobility, self-care, doing usual activities, and having pain/discomfort or feeling very worried/sad, from the EQ-5-DY scale.

There was an observable pattern for four of the five variables from the EQ-5-DY scale: with the exception of self-care, the prevalence of problems 12 months post-index-test was always lowest for the NN group and greatest for the PP group (Figure 3). Moreover, 20.4% of NN and 26.6% of PN met the research definition of the PCC 12-month post-index test (excluding the criteria of a positive test for the former). Children and young people meeting this definition, irrespective of initial test status, had higher scores (i.e., had more problems) on the SDQ with respect to total difficulties (i.e., emotional, conduct, hyperactivity, and peer relationship problems). Again, irrespective of initial test status, children and young people meeting the research definition of PCC had more adverse measures of well-being, fatigue, self-rated health, symptom severity, and impact (each item was analyzed separately, see Supplementary Table S3). 

### 3.4. Comparing Symptom Profiles of the PP Group to the NP and PN Groups

Of the PP group, 24.0% had five-plus symptoms 12 months post-index-text compared to 15.9% of the PN and 19.7% of the NP groups (pchi2-test < 0.001, Supplementary Table S2). Tiredness, sleeping difficulties, shortness of breath, and headaches were more common in the PP group compared to the NP and PN groups (pchi2-test < 0.001). While 2% of the NP and PN groups reported hospital attendance in relation to COVID-19 during the 12-month period, 3.5% of the PP group reported hospital attendance (pchi2-test < 0.001). In all cases, the prevalence of hospital overnight stays was low, varying from 1.0% (78/8060 for PN) to 1.3% (9/684 for PP). In the subsample with information, there was no evidence of a difference in symptom severity or impact comparing the NP and PN groups to the PP group (pMann–Whitney > 0.32 (severity); pMann–Whitney > 0.11 (impact)).

### 3.5. Symptom Profiles by Vaccination Status

Most characteristics 12 months post-index-test (i.e., number of symptoms, scores using the validated instruments for mental health, fatigue) were similar regardless of vaccination status, although there was some variation (in the subsample with data) in symptom severity and impact (Supplementary Table S4).

## 4. Discussion

In our sample of 20,202 children and young people at 12-month follow-up, the prevalence of five-plus symptoms varied between 16–24% for those who had at least one positive test but was 10% for those who never reported a positive test. We found that the most commonly reported symptoms 12 months post-index-test were tiredness, sleeping difficulties, shortness of breath, and headaches. The group that was initially positive and was then reinfected (PP) had more symptoms and a higher prevalence of specific symptoms (e.g., tiredness, sleeping difficulties) compared to the other two infected groups (NP and PN). The prevalence of hospital overnight stays was low in our sample, but the PP group reported hospital attendance in relation to COVID-19 more frequently than the other two infected groups. Perhaps surprisingly, we found that self-rated health was broadly similar 12 months post-testing for all infection status groups. When we operationalised our research definition of PCC [10], 20.4% of the NN group met this definition at 12-month follow-up (minus the need for a positive PCR test) compared to 26.6% of the PN group.

Are these prevalences of 26.6% and 20.4% similar because many of the problems reported are the consequence of a long pandemic rather than directly attributable to viral infection? Are the prevalences similar because 99% of teenagers have now been exposed to SARS-CoV-2 even if they never had a positive test? Or is the excess of 26.6% versus 20.4% a measure of the added burden attributable to PCC, over and above living through a long pandemic? These are challenging questions that have yet to be answered, and the findings should be placed in the context of other studies that face the same challenges of interpretation of findings. Our systematic review, including 55 international studies, with outcomes assessed ≥12 weeks after infection, showed higher pooled estimates of symptoms in SARS-CoV-2 cases for altered/loss of smell/taste, dyspnoea/wheeze, fatigue/weakness, and myalgia (with risk differences ranging from 1% to 4%) [12]. However, none of the included studies separated the sample into four groups, as has been done for the present study. In the systematic review [12], a minority of studies involved follow-up for over 6 months with numbers similar/greater than the present study. Donnachie et al. [28] reported that after 2 years, 7.5–10% of children aged 12–17 were diagnosed with PCC. Similarly, Roessler et al. [29] reported 11,950 PCR-proven cases and 59,750 ‘controls’ aged 0–11 years old, with a mean follow-up time of 236 days. The COVID-19 cohort of children had higher rates of malaise/fatigue/exhaustion, cough, and throat/chest pain, and the 12.6–36.6% incidence rates of these symptoms [29] are of similar magnitude to those experienced more commonly by test-positives at 12-month follow-up in our study. It is difficult to compare our findings to some other large studies from Europe which used different methodologies. The study by Borch [30] utilised a cohort of 15,041 SARS-CoV-2 children and young people (39.1% age 6–17 years) and 15,080 controls. No more than 15% of any age group were followed-up for more than 5 months (i.e., less than 1000 children and young people in total aged 6–17 years old). While 54–75% recovered within 5 months, it is difficult to interpret the data for the sub-group with follow-up over 5 months. Similarly, Kikkenborg Berg et al. reported online surveys on 6630 cases and 21,640 ‘controls’ aged 0–14 years old [31] and 10,997 cases and 3016 controls with a median age of 17.6 years [32]. 13.4% 12–14-year-olds (N = 470) and 16.4% of older adolescents (N = 1085) were followed-up for more than 9 months. Cases had higher odds of at least one symptom lasting more than 3 months (42% vs. 37% 12–14-year-olds; 62% vs. 57% older adolescents), but it is difficult to decipher the data for those with follow-up over 9 months. 

We have published a longitudinal analysis in a smaller subgroup of 5086 children and young people (2909 SARS-CoV-2 Positive; 2177 SARS-CoV-2 Negative at baseline) aged 11–17 who completed questionnaires at both 6 and 12 months after PCR-tests [9]. At all time points, symptoms were more common in test-positive compared to test-negative children and young people, but non-negligible numbers of both test-positive and test-negative children and young people reported adverse symptoms for the first time at six- and 12-months post-test (in particular tiredness, shortness of breath and poor quality of life). This phenomenon of problems arising anew a long time after the original PCR test would contribute to the prevalences we report at 12 months from the larger dataset of 20,202 children and young people. The added value of this manuscript is we are now able to present information not just on ‘initial-negatives’ and ‘initial-positives’ but two other groups who were infected between their index test and the 12-month survey.

Our study has limitations that have previously been highlighted [9]. For example, symptoms at the time of testing may be subject to recall bias because they were reported at the time of first contact with the CLoCk study (at either 3 months, 6 months, or 12 months post-test). However, symptoms 12 months post-test were reported prospectively. It is possible that some children and young people might have been misdiagnosed as SARS-CoV-2 negative and vice-versa. False negatives might be attributable to the timing of the PCR test, swab technique, and assay sensitivity, but false-positive PCR results are rare. Moreover, while we tracked children and young people who had PCR-proven SARS-CoV-2 and self-reported infections after their baseline PCR test, we recognise that (re)infections may have gone undetected. However, the low prevalence of loss of smell/taste among the NN group, at both time of index-testing (retrospective report) and 12 months post-testing, provides some assurance of a low rate of unconfirmed SARS-CoV-2 infections. Collecting information on antibodies would have been ideal, but it was unfeasible and unfunded in a study of this size. The response rate at 12 months post-index test was 8.9% (for the target test-negative group) and 9.6% (for the target test-positive group). Nonetheless, in general, responders included in our analytical sample were similar to the target population, albeit with some differences with respect to sex, deprivation, and region. We also acknowledge that the CLoCk study design may induce selection biases. For example, it may favor those with internet access, and children and young people may be more likely to participate if they have symptoms to report. We have recently developed flexible survey weights to address potential bias and selection issues in the CLoCk study [33] and have demonstrated that previously reported prospective findings from CLoCk (based on a sub-sample of those presented in the current manuscript) [9] are generalisable to the wider population of children and young people in England [33], within the examined age-range (11–17 years). We acknowledge the limitations associated with examining self-reported data, compared to in-person medical interviews; for example, we assessed shortness of breath, headaches, sleeping difficulties, etc., using a single question for each symptom. In-person interviews with CLoCk study participants were not feasible or practical to conduct and in large-scale epidemiological studies such as CLoCk, self-report is an appropriate data collection technique. Nonetheless, we appreciate that some symptoms, such as shortness of breath, might be better assessed by alternative measures. We also acknowledge the issue of floor and ceiling effects (i.e., if the question or validated scale is relatively easy/difficult such that a substantial proportion of respondents obtain either the minimum or maximum scores, then the true extent of their abilities cannot be determined). One advantage of the current report is that we have considered the test results of children and young people in the intervening period between the index test and their 12-month questionnaire, but we cannot state when during the 12-month period the infection occurred. Given it was not possible to distinguish between acute and long-term symptoms in the NP and PP groups, caution is needed in concluding a “dose-response” relationship in long-term symptoms with respect to the number of exposures. Similarly, we cannot state when vaccination occurred during the 12-month period and are unable to determine the chronology of (re)infection and vaccination. This may account for our observation that there was no difference between the groups of children and young people who did and did not have a vaccine in relation to symptoms experienced, quality of life, mental health, well-being, or fatigue. Finally, we appreciate that as researchers, we want to ask as much as possible of the children and young people to enable varied and extensive analysis addressing as many specific research questions as possible. Hence, our initial draft questionnaire took more than one hour to complete. However, in our pilot study, children and young people said they would be willing to only spend a maximum of 20 minutes completing the survey. Therefore, compromises had to be made. As such, while our data is unique and wide-ranging and adds value to the literature, it is limited in terms of the depth of information available.

## 5. Conclusions

Of 11–17 year-olds, 24.0% with more than one positive test, have five-plus symptoms at 12 months compared to 10.2% of those who originally tested negative with no later infection. Following the pandemic, many young people, particularly those that have had multiple SARS-CoV-2 positive tests, experience a range of symptoms that warrant consideration and potential investigation and intervention.

## Figures and Tables

**Table 1:** Demographics of the target population and participants included in the 12-month sample.

	Target Test-Negative Population (*n* = 128,161)	Initial-Negatives (NN) (*n* = 7474)	Negatives and Infected (NP) (*n* = 3948)	Target Test-Positive Population (*n* = 91,014)	Initial-Positives (PN) (*n* = 8060)	Positive-Reinfected (PP) (*n* = 684)
		Response rate: 8.9%			Response rate: 9.6%	
Sex						
Female	67,194 (52.4%)	4644 (62.1%)	2466 (62.5%)	48,042 (52.8%)	5023 (62.3%)	420 (61.4%)
Male	60,967 (47.6%)	2830 (37.9%)	1482 (37.5%)	42,972 (47.2%)	3037 (37.7)	264 (38.6%)
Age (years)						
11–14	65,951 (51.5%)	3481 (46.6%)	2271 (57.5%)	46,106 (50.7%)	3892 (48.3%)	369 (54.0%)
15–17	62,210 (48.5%)	3993 (53.4%)	1677 (42.5%)	44,908 (49.3%)	4168 (51.7%)	315 (46.0%)
Ethnicity	Not recorded			Not recorded		
White		5529 (74.0%)	3141 (79.6%)		6001 (74.5%)	529 (77.3%)
Asian, Asian British		1143 (15.3%)	420 (10.6%)		1235 (15.3%)	90 (13.2%)
Mixed		421 (5.6%)	224 (5.7%)		422 (5.2%)	34 (5.0%)
Black, African, Caribbean		245 (3.3%)	92 (2.3%)		222 (2.8%)	19 (2.8%)
Other		94 (1.3%)	59 (1.5%)		140 (1.7%)	7 (1.0%)
Unknown		42 (0.6%)	12 (0.3%)		40 (0.5%)	5 (0.7%)
Region						
East Midlands	7861 (6.1%)	401 (5.4%)	208 (5.3%)	6248 (6.9%)	519 (6.4%)	54 (7.9%)
East of England	24,919 (19.4%)	1906 (25.5%)	1232 (31.2%)	13,982 (15.4%)	1552 (19.3%)	127 (18.6%)
London	27,156 (21.2%)	1832 (24.5%)	831 (21.1%)	19,144 (21.0%)	1782 (22.1%)	161 (23.5%)
North East England	4825 (3.8%)	199 (2.7%)	84 (2.1%)	3788 (4.2%)	284 (3.5%)	28 (4.1%)
North West England	17,950 (14.0%)	704 (9.4%)	300 (7.6%)	13,339 (14.7%)	872 (10.8%)	67 (9.8%)
South East England	18,251 (14.2%)	1236 (16.5%)	730 (18.5%)	13,316 (14.6%)	1431 (17.8%)	100 (14.6%)
South West England	4563 (3.6%)	240 (3.2%)	129 (3.3%)	3576 (3.9%)	356 (4.4%)	44 (6.4%)
West Midlands	12,881 (10.0%)	600 (8.0%)	261 (6.6%)	9800 (10.8%)	736 (9.1%)	57 (8.3%)
Yorkshire and the Humber	9755 (7.6%)	356 (4.8%)	173 (4.4%)	7821 (8.6%)	528 (6.6%)	46 (6.7%)
IMD quintile ^a^						
1 (most deprived)	31,116 (24.3%)	1121 (15.0%)	484 (12.3%)	22,963 (25.2%)	1182 (14.7%)	118 (17.3%)
2	25,744 (20.1%)	1306 (17.5%)	639 (16.2%)	19,013 (20.9%)	1435 (17.8%)	123 (18.0%)
3	23,423 (18.3%)	1435 (19.2%)	740 (18.7%)	16,453 (18.1%)	1506 (18.7%)	134 (19.6%)
4	23,725 (18.5%)	1711 (22.9%)	907 (23.0%)	16,271 (17.9%)	1781 (22.1%)	142 (20.8%)
5 (least deprived)	24,153 (18.8%)	1901 (25.4%)	1178 (29.8%)	16,314 (17.9%)	2156 (26.8%)	167 (24.4%)

^a^ IMD: Index of Multiple Deprivation. The Index of Multiple Deprivation (IMD) was calculated from the children and young peoples’ small local area level-based geographic hierarchy (lower super output area) at the time of the first questionnaire and used as a proxy for socioeconomic status. We report IMD quintiles from most (quintile 1) to least (quintile 5) deprived.

## Data Availability

Data are not publicly available. All requests for data will be reviewed by the Children & Young People with Long COVID-19 (CLoCk) study team to verify whether the request is subject to any intellectual property or confidentiality obligations. Requests for access to the participant-level data from this study can be submitted via email to Clock@phe.gov.uk with detailed proposals for approval. A signed data access agreement with the CLoCk team is required before accessing shared data. Code is not made available as we have not used custom code or algorithms central to our conclusions.

## Supplementary Materials

The following supporting information can be downloaded at: https://www.mdpi.com/article/10.3390/children10071227/s1, Table S1: Characteristics at baseline of children and young people by SARS-CoV-2 and vaccination status by 12 months; Table S2: Reported symptoms N (%), and self-rated health, by SARS-CoV-2 status, at time of index test and 12 months post-test; Table S3. Characteristics 12 months post-index-test by SARS-CoV-2 status, stratified by PCC at 12 months (Mean (SD)); Table S4. Characteristics of children and young people 12 months post-index-test by SARS-CoV-2 and vaccination status at 12 months (N (%) or Mean (SD)).
