# Supplementary material for: Symptom Profiles of Children and Young People 12 Months after SARS-CoV-2 Testing: A National Matched Cohort Study (The CLoCk Study)
Source: Children (Basel). 2023 Jul 14;10(7):1227. doi: 10.3390/children10071227 (PMC10377812; doi:10.3390/children10071227)
Supplement: Supplementary file 1 [file children-10-01227-s001.zip › children-2456686-supplementary.pdf]

Supplementary Table S1. Characteristics at baseline of CYP by SARS-CoV-2 and vaccination status[a] by 12 months.

	**Initial-negatives (NN)** (N=7,474)		**Negative & infected (NP)** (N=3,948)		**Initial-positives (PN)** (N= 8,060)		**Positive & reinfected (PP)** (N= 684)	
	**Vaccine: No n (%)**	**Vaccine: Yes n (%)**	**Vaccine: No n (%)**	**Vaccine: Yes n (%)**	**Vaccine: No n (%)**	**Vaccine: Yes n (%)**	**Vaccine: No n (%)**	**Vaccine: Yes n (%)**
**Response Rate**	1,535 (20.6%)	5,928 (79.4%)	1,310 (33.3%)	2,628 (66.7%)	1,930 (24.0%)	6,113 (76.0%)	257 (37.6%)	427 (62.4%)
**Sex**								
Male	644 (22.8%)	2,182 (77.2%)	516 (34.9%)	962 (65.1%)	794 (26.2%)	2,233 (73.8%)	118 (44.7%)	146 (55.3%)
Female	891 (19.2%)	3,746 (80.8%)	794 (32.3%)	1,666 (67.7%)	1,136 (22.7%)	3,880 (77.4%)	139 (33.1%)	281 (66.9%)
**Age (years)**								
11-14	1,057 (30.4%)	2,417 (69.6%)	998 (44.0%)	1,268 (56.0%)	1,310 (33.8%)	2,570 (66.2%)	184 (49.9%)	185 (50.1%)
15-17	478 (12.0%)	3,511 (88.0%)	312 (18.7%)	1,360 (81.3%)	620 (14.9%)	3,543 (85.1%)	73 (23.2%)	242 (76.8%)
**Ethnicity**								
White	974 (17.7%)	4,545 (82.3%)	1,021 (32.6%)	2,111 (67.4%)	1,275 (21.3%)	4,715 (78.7%)	180 (34.0%)	349 (66.0%)
Asian or Asian British	325 (28.4%)	818 (71.6%)	148 (35.2%)	272 (64.8%)	365 (29.7%)	865 (70.3%)	41 (45.6%)	49 (54.4%)
Mixed	109 (25.9%)	313 (74.1%)	74 (33.0%)	150 (67.0%)	120 (28.4%)	302 (71.6%)	15 (44.1%)	19 (55.9%)
Black, African or Caribbean	85 (34.8%)	159 (65.2%)	42 (46.2%)	49 (53.9%)	109 (49.1%)	113 (50.9%)	11 (57.9%)	8 (42.1%)
Other	25 (26.6%)	69 (73.4%)	20 (33.9%)	39 (66.1%)	40 (28.6%)	100 (71.4%)	6 (85.7%)	1 (14.3%)
Prefer not to say	17 (40.5%)	25 (59.5%)	5 (41.7%)	7 (58.3%)	21 (53.9%)	18 (46.2%)	4 (80.0%)	1 (20.0%)
**Region**								
East Midlands	77 (19.3%)	323 (80.8%)	60 (29.1%)	146 (70.9%)	102 (19.7%)	416 (80.3%)	16 (29.6%)	38 (70.4%)
East of England	293 (15.4%)	1,611 (84.6%)	357 (29.1%)	872 (71.0%)	306 (19.8%)	1,242 (80.2%)	41 (32.3%)	86 (67.7%)

	**Initial-negatives** (N=7,474)		**Negative & infected** (N=3,948)		**Initial-positives** (N= 8,060)		**Positive & reinfected** (N= 684)	
	**Vaccine: No n (%)**	**Vaccine: Yes n (%)**	**Vaccine: No n (%)**	**Vaccine: Yes n (%)**	**Vaccine: No n (%)**	**Vaccine: Yes n (%)**	**Vaccine: No n (%)**	**Vaccine: Yes n (%)**
**Response Rate**	1,535 (20.6%)	5,928 (79.4%)	1,310 (33.3%)	2,628 (66.7%)	1,930 (24.0%)	6,113 (76.0%)	257 (37.6%)	427 (62.4%)

**Region**								
London	378 (20.6%)	1,453 (79.4%)	293 (35.3%)	536 (64.7%)	444 (25.0%)	1,331 (75.0%)	70 (43.5%)	91 (56.5%)
North East	52 (26.3%)	146 (73.7%)	33 (39.3%)	51 (60.7%)	75 (26.4%)	209 (73.6%)	8 (28.6%)	20 (71.4%)
North West	258 (36.8%)	443 (63.2%)	145 (48.5%)	154 (51.5%)	285 (32.7%)	587 (67.3%)	30 (44.8%)	37 (55.2%)
South East	160 (12.9%)	1,076 (87.1%)	229 (31.4%)	500 (68.6%)	257 (18.0%)	1,172 (82.0%)	32 (32.0%)	68 (68.0%)
South West	44 (18.3%)	196 (81.7%)	36 (27.9%)	93 (72.1%)	78 (22.0%)	277 (78.0%)	12 (27.3%)	32 (72.7%)
West Midlands	157 (26.3%)	441 (73.8%)	85 (32.7%)	175 (67.3%)	199 (27.1%)	535 (72.9%)	30 (52.6%)	27 (47.4%)
Yorkshire and the Humber	116 (32.7.%)	239 (67.3%)	72 (41.6%)	101 (58.4%)	184 (34.9%)	344 (65.2%)	18 (39.1%)	28 (60.9%)
**IMD quintile**								
1 (most deprived)	426 (38.0%)	694 (62.0%)	218 (45.1%)	265 (54.9%)	457 (38.8%)	722 (61.2%)	54 (45.8%)	64 (54.2%)
2	308 (23.6%)	995 (76.4%)	242 (37.9%)	397 (62.1%)	410 (28.7%)	1,021 (71.4%)	54 (43.9%)	69 (56.1%)
3	267 (18.6%)	1,166 (81.4%)	243 (32.9%)	496 (67.1%)	339 (22.6%)	1,161 (77.4%)	45 (33.6%)	89 (66.4%)
4	278 (16.3%)	1,433 (83.8%)	273 (30.2%)	631 (69.8%)	372 (20.9%)	1,408 (79.1%)	52 (36.6%)	90 (63.4%)
5 (least deprived)	256 (13.5%)	1,640 (86.5%)	334 (28.5%)	839 (71.5%)	352 (16.4%)	1,801 (83.7%)	52 (31.1%)	115 (68.9%)

[a]Those who did not respond to the vaccine question (n=38) were excluded.

Supplementary Table S2. Reported symptoms N(%), and self-rated health[a], by SARS-CoV-2 status, at time of index test and 12 months post-test.

	At time of index test[b]				*12 months post-test*			
	Initial-negatives (NN) n =7,474	Negative & infected (NP) n=3,948	Initial-positives (PN) n=8,060	Positive & re-infected (PP) n=684	Initial-negatives (NN) n = 7,474	Negative & infected (NP) n=3,984	Initial-positives (PN) n= 8,060	Positive & re-infected (PP) n=684
**Number of reported symptoms**								
0 symptom	6,731 (90.1%)	2,570 (65.1%)	5,037 (62.5%)	382 (55.9%)	3,740 (50.0%)	1,281(32.5%)	3,117 (38.7%)	177 (25.9%)
1 symptom	70 (0.9%)	63 (1.6%)	249 (3.1%)	28 (4.1%)	1,357 (18.2%)	748 (19.0%)	1,444 (17.9%)	130 (19.0%)
2 symptoms	93 (1.2%)	92 (2.3%)	300 (3.7%)	19 (2.8%)	756 (10.1%)	525 (13.3%)	962 (11.9%)	79 (11.6%)
3 symptoms	110 (1.5%)	131 (3.3%)	341 (4.2%)	28 (4.1%)	507 (6.8%)	356 (9.0%)	720 (8.9%)	67 (9.8%)
4 symptoms	84 (1.1%)	151 (3.8%)	359 (4.5%)	26 (3.8%)	351 (4.7%)	259 (6.6%)	530 (6.6%)	67 (9.8%)
≥5 symptoms	386 (5.2%)	941 (23.8%)	1,774 (22.0%)	201 (29.4%)	763 (10.2%)	779 (19.7%)	1,287 (16.0%)	164 (24.0%)
**Specific symptoms**								
Fever	311 (4.2%)	729 (18.5%)	1,468 (18.2%)	146 (21.4%)	151 (2.0%)	109 (2.8%)	168 (2.1%)	38 (5.6%)
Chills	249 (3.3%)	665 (16.8%)	1,224 (15.2%)	133 (19.4%)	655 (8.8%)	452 (11.5%)	928 (11.5%)	97 (14.2%)
Persistent cough	361 (4.8%)	730 (18.5%)	1,205 (15.0%)	145 (21.2%)	438 (5.9%)	348 (8.8%)	499 (6.2%)	80 (11.7%)
Tiredness	363 (4.9%)	893 (22.6%)	1,821 (22.6%)	186 (27.2%)	2,383 (31.9%)	1,756 (44.5%)	3,388 (42.0%)	369 (54.0%)
Shortness of breath	147 (2.0%)	424 (10.7%)	820 (10.2%)	89 (13.0%)	956 (12.8%)	991 (25.1%)	1,787 (22.2%)	197 (28.8%)
Loss of smell	135 (1.8%)	741 (18.8%)	1,777 (22.0%)	158 (23.1%)	106 (1.4%)	641 (16.2%)	582 (7.2%)	75 (11.0%)
Unusually hoarse voice	161 (2.2%)	268 (6.8%)	368 (4.6%)	50 (7.3%)	168 (2.3%)	132 (3.3%)	143 (1.8%)	29 (4.2%)
Unusual chest pain	135 (1.8%)	349 (8.8%)	627 (7.8%)	68 (9.9%)	295 (4.0%)	303 (7.7%)	508 (6.3%)	60 (8.8%)
Unusual abdominal pain	82 (1.1%)	178 (4.5%)	284 (3.5%)	46 (6.7%)	188 (2.5%)	157 (4.0%)	333 (4.1%)	40 (5.9%)
Diarrhoea	87 (1.2%)	196 (5.0%)	340 (4.2%)	42 (6.1%)	127 (1.7%)	96 (2.4%)	194 (2.4%)	19 (2.8%)
Headaches	452 (6.1%)	1,013 (25.7%)	2,074 (25.7%)	223 (32.6%)	884 (11.8%)	745 (18.9%)	1,349 (16.7%)	169 (24.7%)
Confusion, disorientation or drowsiness	102 (1.4%)	253 (6.4%)	553 (6.9%)	55 (8.0%)	264 (3.5%)	223 (5.7%)	433 (5.4%)	50 (7.3%)

Unusual eye-soreness	83 (1.1%)	276 (7.0%)	437 (5.4%)	57 (8.3%)	292 (3.9%)	261 (6.6%)	426 (5.3%)	53 (7.8%)
Skipping meals	169 (2.3%)	489 (12.4%)	888 (11.0%)	93 (13.6%)	526 (7.0%)	420 (10.6%)	737 (9.1%)	78 (11.4%)
Dizziness or light-headedness	235 (3.1%)	623 (15.8%)	1,171 (14.5%)	126 (18.4%)	573 (7.7%)	491 (12.4%)	973 (12.1%)	134 (19.6%)
Sore throat	533 (7.1%)	985 (25.0%)	1,743 (21.6%)	197 (28.8%)	559 (7.5%)	424 (10.7%)	568 (7.1%)	109 (15.9%)
	At time of index test[b]				*12 months post-test*			
	Initial-negatives (n =7,474)	Negative & infected (n=3,948)	Initial-positives (n=8,060)	Positive & re-infected (n=684)	Initial-negatives (n = 7,474)	Negative & infected (n=3,984)	Initial-positives (n= 8,060)	Positive & re-infected (n=684)
Unusual strong muscle pains	116 (1.6%)	374 (9.5%)	735 (9.1%)	93 (13.6%)	208 (2.8%)	210 (5.3%)	361 (4.5%)	48 (7.0%)
Earache or ringing in ears	118 (1.6%)	219 (5.6%)	335 (4.2%)	49 (7.2%)	351 (4.7%)	231 (5.9%)	534 (6.6%)	52 (7.6%)
Raised welts on skin or swelling	18 (0.2%)	40 (1.0%)	78 (1.0%)	12 (1.8%)	54 (0.7%)	41 (1.0%)	102 (1.3%)	9 (1.3%)
Red/purple sores/blisters on feet	12 (0.2%)	14 (0.4%)	70 (0.9%)	3 (0.4%)	47 (0.6%)	32 (0.8%)	65 (0.8%)	3 (0.4%)
Sleeping difficulties[c]	N/A	N/A	N/A	N/A	1,733 (23.2%)	1,173 (29.7%)	2,229 (27.7%)	242 (35.4%)
Other	42 (0.6%)	108 (2.7%)	246 (3.1%)	28 (4.1%)	239 (3.2%)	266 (6.7%)	398 (4.9%)	50 (7.3%)
Self-rated health[a]	90 (80, 100)	90 (80, 95)	90 (85,100)	90 (85, 100)	90 (80, 95)	85 (75,95)	90 (75, 95)	85 (75,95)

[a]Reported as median(IQR), scored on a scale of 0 (worst) to 100 (best).
[b]Retrospectively reported, see methods for details.
[c]Sleeping difficulties only included at 12 months post-test questionnaire.

Supplementary Table S3. Characteristics 12 months post index-test by SARS-CoV-2 status, stratified by PCC[a] at 12 months (Mean (SD)).

	SARS-CoV-2 Test Negatives (NN) N=7,474		SARS-CoV-2 Test Positives (PN) N=8,060	
	Not meeting PCC definition at 12 months (n=5,951)	Meeting PCC definition at 12 months (n=1,523)	Not meeting PCC definition at 12 months (n=5,917)	Meeting PCC definition at 12 months (n=2,143)
**Prevalence**		20.4%		26.6%
**SDQ[b]**				
Total Difficulties	10.0 (5.7)	16.8(6.0)	9.5 (5.5)	16.0 (6.2)
Emotional symptoms	3.1 (2.3)	5.8 (2.4)	2.9 (2.3)	5.7 (2.5)
Conduct problems	1.4 (1.4)	2.3 (1.8)	1.4 (1.4)	2.1 (1.8)
Hyperactivity/inattention	3.6 (2.5)	5.5 (2.5)	3.5 (2.4)	5.3 (2.6)
Peer relationship problem	1.9 (1.7)	3.1 (2.0)	1.8 (1.6)	2.9 (2.0)
**SWEMWBS**	21.9 (4.1)	18.6 (3.3)	22.2 (4.1)	19.2 (3.6)
**Chalder fatigue scale**	11.9 (4.3)	17.7 (5.7)	12.2 (4.2)	18.0 (5.6)
**Self-rated health[b,c]**	90 (80,100)	75 (60,85)	90 (85, 95)	75 (60,85)
**Symptom severity[d]**	50 (20,60)	60 (30,80)	50 (30,70)	50 (40,70)
**Symptom impact[e]**	30 (10,60)	60 (30,80)	30 (10,60)	50 (20,70)

Note. SDQ = Strengths and Difficulties Questionnaire; SWEMBS = Short Warwick-Edinburgh Mental Wellbeing Scale.
A higher SDQ score indicates more problems; a higher SWEMWBS score indicates better mental well-being; a higher fatigue score is more severe.
[a]Using data from the questionnaire on symptoms and the EQ-5D-Y scale at the time of the questionnaire (i.e., approximately 12-months after the index PCR-test), PCC was operationalized as having at least 1 symptom and experiencing some/a lot of problems with respect to mobility, self-care, doing usual activities or having pain/discomfort or feeling very worried/sad. For test-negatives we excluded the need for a positive PCR test. We cannot state with certainty when during the 12-month period the subsequent infections occurred (some of this information was based on self-report), therefore we did not operationalise the PCC research definition for the NP or PP groups. Number of symptoms and the EQ-5D-Y scale at 12 months not shown in this table as they are part of the definition of PCC.
[b]Missing data for SDQ (n=1 who tested positive); missing data for Self-rated health (n=2 ; 1 tested positive and 1 tested negative).
[c]reported as median(IQR), scored on a scale of 0 (worst) to 100 (best).
[d]Symptom severity sub-sample N=621, scale is 0 (not severe at all) to 100 (extremely severe).
[e]Symptom impact sub-sample N=618, a scale is 0 (no impact) to 100 (extreme impact).

Supplementary Table S4. Characteristics of CYP 12 months post index-test by SARS-CoV-2 and vaccination status at 12 months (N(%) or Mean(SD)).

	Initial test-negatives (NN) N=7,463		Negative & infected (NP) N=3,938		Initial test-positives (PN) N=8,043		Positive-reinfected (PP) N=684	
	Vaccine: No (n=1,536)	Vaccine: Yes (n=5,927)	Vaccine: No (n=1,310)	Vaccine: Yes (n=2,628)	Vaccine: No (n=1,931)	Vaccine: Yes (n=6,112)	Vaccine: No (n=258)	Vaccine: Yes (n=426)
**Number of symptoms**								
0 symptoms	740 (48.2%)	2,993 (50.5%)	405 (30.9%)	874 (33.3%)	709 (36.7%)	2,400 (39.3%)	64 (24.8%)	113 (26.5%)
1 symptom	261 (17.0%)	1,095 (18.5%)	260 (19.8%)	487 (18.5%)	331 (17.1%)	1,111 (18.2%)	52 (20.2%)	78 (18.3%)
2 symptoms	153 (10.0%)	602 (10.1%)	165 (12.6%)	358 (13.6%)	239 (12.4%)	722 (11.8%)	26 910.1%)	53 (12.4%)
3 symptoms	104 (6.8%)	402 (6.7%)	116 (8.8%)	239 (9.1%)	190 (9.8%)	529 (8.7%)	30 (11.6%)	37 (8.7%)
4 symptoms	79 (5.1%)	272 (4.6%)	92 (7.0%)	167 (6.4%)	130 (6.7%)	400 (6.5%)	25 (9.7%)	42 (9.8%)
≥5 symptoms	199 (12.9%)	563 (9.5%)	272 (20.8%)	503 (19.1%)	332 (17.2%)	950 (15.5%)	61 (23.6%)	103 (24.2%)
**EQ5DY**								
Some/lots of mobility problems	97 (6.3%)	304 (5.1%)	94 (7.2%)	163 (6.2%)	136 (7.0%)	353 (5.8%)	17 (6.6%)	41 (9.6%)
Some/lots of self-care problems	94 (6.1%)	280 (4.7%)	54 (4.1%)	116 (4.4%)	75 (3.9%)	242 (4.0%)	14 (5.4%)	27 (6.3%)
Some/lots of problems with usual activities	239 (15.6%)	757 (12.8%)	267 (20.4%)	471 (17.9%)	352 (18.2%)	911 (14.9%)	53 (20.5%)	86 (20.2%)
Some/lots of pain discomfort	262 (17.1%)	879 (14.8%)	307 (23.4%)	524 (19.9%)	405 (21.0%)	1,067 (17.5%)	58 (22.5%)	102 (23.9%)
Very worried, sad/unhappy	107 (7.0%)	496 (8.4%)	101 (7.7%)	239 (9.1%)	165(8.5%)	542 (8.9%)	19 (7.4%)	46 (10.8%)
**SDQ**[a]								
Total Difficulties	11.2 (6.7)	11.5 (6.3)	11.3 (6.6)	11.4 (6.4)	11.3 (6.7)	11.3 (6.3)	11.2 (6.7)	11.7 (6.3)
Emotional symptoms	3.3 (2.6)	3.7 (2.6)	3.6 (2.6)	3.7 (2.7)	3.5 (2.6)	3.7 (2.6)	3.4 (2.5)	3.9 (2.7)
Conduct problems	1.8 (1.7)	1.6 (1.5)	1.7 (1.7)	1.6 (1.6)	1.7 (1.6)	1.5 (1.5)	1.8 (1.8)	1.6 (1.6)
Hyperactivity/inattention	3.9 (2.7)	4.0 (2.6)	4.0 (2.7)	4.1 (2.6)	3.9 (2.6)	4.0 (2.6)	3.8 (2.6)	4.2 (2.6)

Peer relationship problem	2.1 (1.8)	2.2 (1.9)	1.9 (1.8)	2.0 (1.8)	2.1 (1.8)	2.0 (1.8)	2.0 (2.1)	2.0 (1.8)
	Initial test-negatives (N=7,463)		Negative & infected (N=3,938)		Initial test-positives (N=8,043)		Positive-reinfected (N=684)	
**SWEMBS**	21.5 (4.4)	21.2 (4.1)	21.5 (4.4)	21.3 (4.2)	21.6 (4.5)	21.4 (4.1)	21.6 (4.6)	20.8 (4.0)
**Chalder fatigue scale**	12.6 (5.8)	13.2 (5.0)	13.5 (5.3)	14.1 (5.2)	13.6 (5.5)	13.8 (5.2)	13.8 (5.5)	14.6 (5.4)
**Self-rated health**[b]	90 (80,100)	90 (80,95)	85 (75,95)	85 (75, 95)	90 (75,95)	90 (75,95)	85 (75,95)	85 (70,95)
**Symptom severity**[c]	50 (30,70)	50 (30,70)	50 (40,70)	50 (30,70)	60 (30,70)	50 (30,60)	40 (20,70)	50 (30,60)
**Symptom impact**[d]	30 (0,60)	50 (20,70)	50 (20,70)	50 (20,70)	50 (10,80)	40 (10,60)	50 (10,80)	40 (10,65)

Note: Those who did not respond to the vaccine question (n=38) were excluded.

[a]Missing data for SDQ (n=1 in 'initial-positives').

[b]Reported as median(IQR), scored on a scale of 0 (worst) to 100 (best); missing data for Self-rated health (n=2 ; 1 in "initial-positives" and 1 in "initial-negatives").

[c]Symptom severity sub-sample N=621, scale is 0 (not severe at all) to 100 (extremely severe).

[d]Symptom impact sub-sample N=618, a scale is 0 (no impact) to 100 (extreme impact.
